# Engineering Emulsion Gels as Functional Colloids Emphasizing Food Applications: A Review

**DOI:** 10.3389/fnut.2022.890188

**Published:** 2022-05-17

**Authors:** Abdullah, Lang Liu, Hafiz Umer Javed, Jie Xiao

**Affiliations:** ^1^Guangdong Provincial Key Laboratory of Functional Food Active Substances, College of Food Sciences, South China Agricultural University, Guangzhou, China; ^2^School of Chemistry and Chemical Engineering, Zhongkai University of Agricultural and Engineering, Guangzhou, China

**Keywords:** biopolymer-based colloids, emulsion gels, delivery systems, functional material, gel-body interactions, food applications

## Abstract

Gels are functional materials with well-defined structures (three-dimensional networks) assembled from the dispersed colloids, and capable of containing a large amount of water, oil, or air (by replacing the liquid within the gel pores), known as a hydrogel, oleogel, and aerogel, respectively. An emulsion gel is a gelled matrix filled with emulsion dispersion in which at least one phase, either continuous phase or dispersed phase forms spatial networks leading to the formation of a semisolid texture. Recently, the interest in the application of gels as functional colloids has attracted great attention in the food industry due to their tunable morphology and microstructure, promising physicochemical, mechanical, and functional properties, and superior stability, as well as controlled release, features for the encapsulated bioactive compounds. This article covers recent research progress on functional colloids (emulsion gels), including their fabrication, classification (protein-, polysaccharide-, and mixed emulsion gels), and properties specifically those related to the gel-body interactions (texture perception, digestion, and absorption), and industrial applications. The emerging applications, including encapsulation and controlled release, texture design and modification, fat replacement, and probiotics delivery are summarized. A summary of future perspectives to promote emulsion gels' use as functional colloids and delivery systems for scouting potential new applications in the food industry is also proposed. Emulsion gels are promising colloids being used to tailor breakdown behavior and sensory perception of food, as well as for the processing, transportation, and targeted release of food additives, functional ingredients, and bioactive substances with flexibility in designing structural and functional parameters.

## Introduction

A gel is an advanced material possessing three-dimensional (3D) networks with the ability to incorporate large amount of water (hydrogel), oil (oleogel), or air (aerogel), due to its spatial structure and unique properties, including high surface area, porosity, and loading capacity (1, 2). A gel can also be defined as “an intermediate (semisolid) product between a solid and a liquid possessing both elasticity and viscosity characteristics” (3). An emulsion gel is also known as emulsion-filled gel or emulgel is “an emulsion dispersion filled gel matrix, wherein at least one phase either continuous phase or dispersed phase of emulsion forms the 3D network structure leading to the gel formation”. These gels possess superior stabilities against chemical reactions (e.g., hydrolysis and oxidation), physical processes (e.g., phase inversion and/or separation), and environmental changes such as pH, temperature, and ionic strength (4–8) compared to traditional emulsions, which tend to break down with time by gravitational separation, droplets aggregation, and Ostwald ripening (1, 9–11).

**Figure 1:**
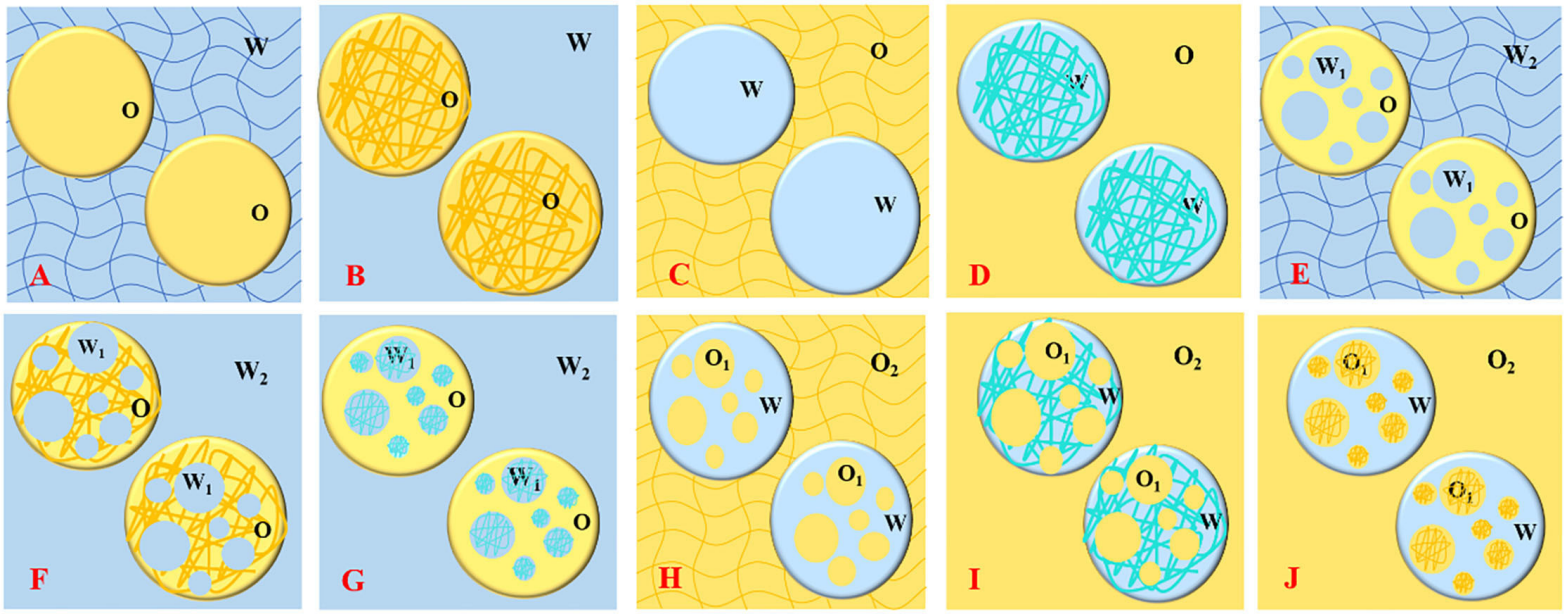
Schematic representation of different types of emulsion gels; oil/hydrogel **(A)**, oleogel/water **(B)**, water/oleogel **(C)**, hydrogel/oil **(D)**, water/oil/hydrogel **(E)**, water/oleogel/water **(F)**, hydrogel/oil/water **(G)**, oil/water/oleogel **(H)**, oil/hydrogel/oil **(I)**, and oleogel/water/oil **(J)**.

**Figure 2:**
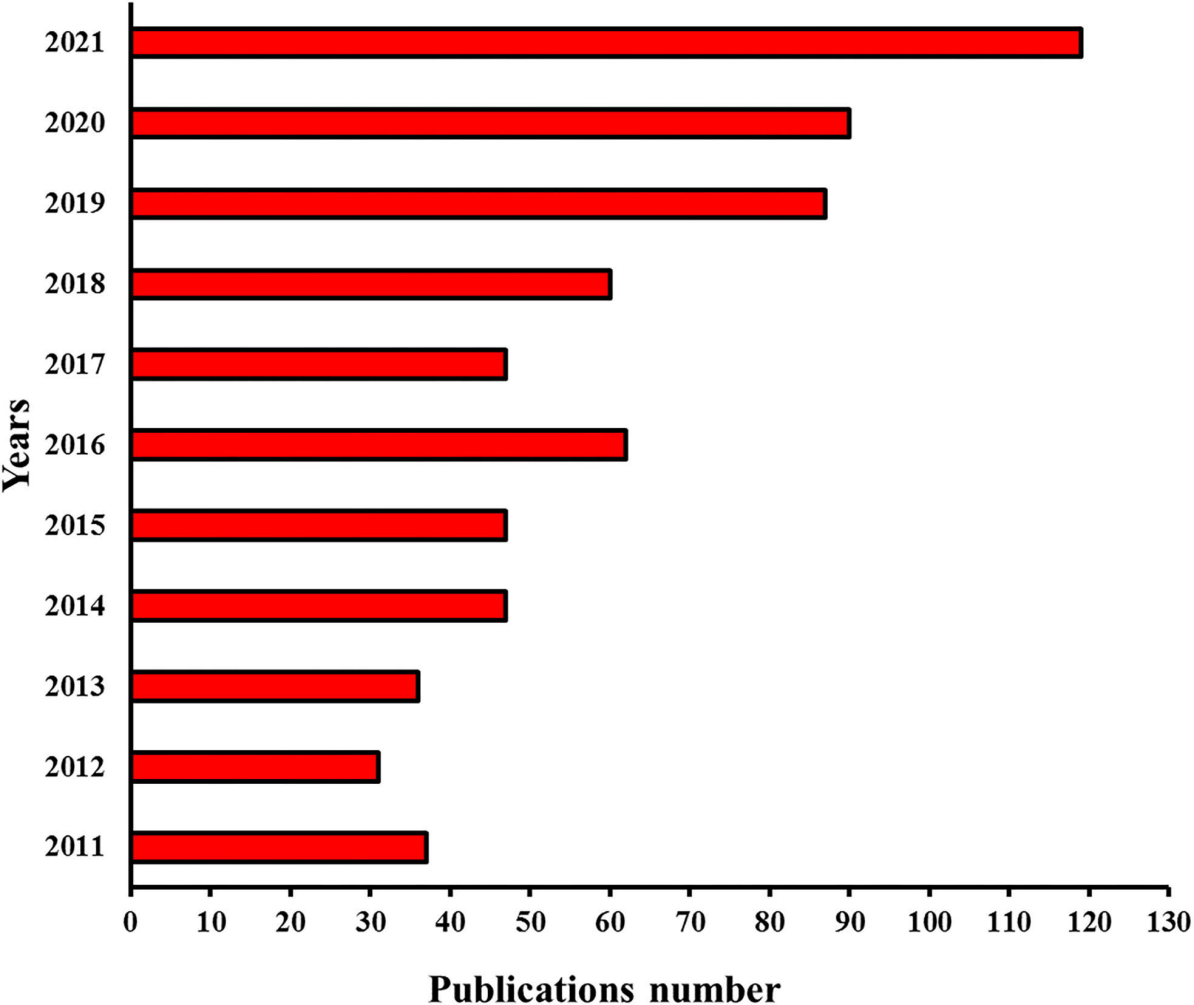
Publications per year during the last 10 years (2011-2021) were analyzed by “Sci-finder” using “emulsion gel” as the keyword for searching.

The biopolymers (e.g., polysaccharides and proteins) based emulsion gels produced by different gelation methods and coupled with a wide range of functionalities had the capability of forming complex microstructures such as single continuous phase-, double continuous phase-, uniform continuous-, and nonhomogeneous as well as several other gelled systems (Figure 1) (12, 13), making them diverse biomaterial and efficient delivery vehicles for versatile industrial applications. Functional food additives, bioactive phytochemicals, essential oils, and lipophilic compounds including carotenoids, phenolic acids, flavonoids, stilbenes, vitamins, and unsaturated fatty acids exhibit health-promoting characteristics but have difficulty being incorporated into food matrixes due to their low chemical stability, limited water solubility and dispersibility, as well as poor cell adsorption (12, 14–18). During the past 10 years (Figure 2), emulsion gels have emerged as a promising biomaterial with desirable features and flexible fabrication potential to be employed for the protection and transportation of health-promoting functional ingredients and designing heathier formulations with improved desired sensorial textures, digestion, bioaccessibility, and bioavailability (8, 12, 17, 19, 20).

In recent years, many studies have reported the potential of emulsion gels to effectively encapsulate, protect, and targeted release of functional ingredients and nutraceuticals against adverse environmental conditions by modifying their dispersibility and stability in the food, controlling their release time and rate, as well as improving bioavailability. To date, emerging applications of emulsion gels to encapsulate and deliver hydrophilic and lipophilic nutraceuticals (7, 21, 22), controlled release of bioactives (12, 23), low fat foods with reduced lipolysis (24, 25), reducing fat, sugar, and salt in foods (26–28), probiotics delivery in the gastrointestinal tract with improved viability (29), desired sensorial textures with improved physical stability (30, 31), structuring plant-oils as animal-fat replacer and substitute of partially hydrogenated oils (32–34), and designing complex food structures (textures, shapes, and nutritional contents) using 3D printing (35) have attracted tremendous attentions for designing safer, healthier, and sustainable food products.

**Figure 3:**
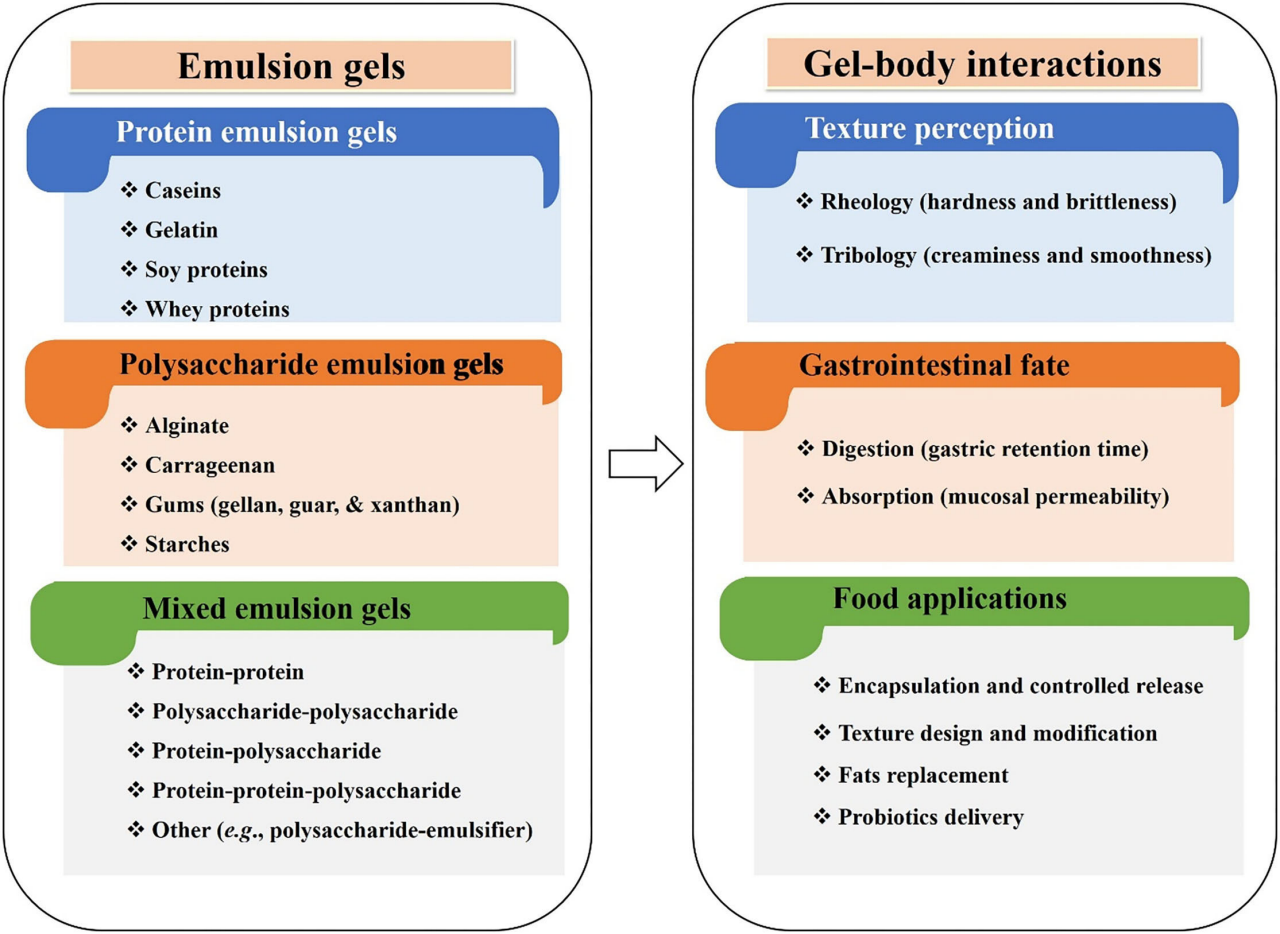
An overview of emulsion gels, indicating their classification, gel-body interactions (texture perception, digestion, and absorption), and emerging industrial food applications.

This review summarizes the latest developments in engineering biopolymers (e.g., polysaccharides and proteins) based food gels, emphasizing their food applications. The gels fabrication, their classification according to the composition of the gel matrix (protein-, polysaccharide-, and mixed emulsion gels), and properties, specifically those related to the gel-body interactions (texture perception, digestion, and absorption), are all discussed (Figure 3). The emerging industrial applications, including encapsulation and controlled release, texture design and modification, fat replacement, and probiotics delivery are highlighted. A summary of conclusions and future perspectives for scouting potential new applications of emulsion gels in the food industry is also presented.

## Emulsion Gels: Fabrication and Classification

The formation of an emulsion gel takes place *via* gelation of the following: (i) continuous phase or (ii) dispersed phase in the precursor solution through different methods, including emulsification, heating, heating and cooling, enzymatic treatment, pH adjustment, salt induced, *etc*. It has been established that gels formed *via* gelation usually had superior stability against environmental stresses due to their strong networks (3D) developed by the interconnected biopolymer molecules (1, 12). The gelation process that leads to the formation of gel matrix plays a key role in determining the final properties (physicochemical, mechanical, and functional) of emulsion gels. Briefly, the biopolymer type and concentration, processing conditions (pH, temperature, and ionic strength), Pickering particles, such as emulsifiers (size, wettability, surface charge, and amount), and cooling temperature, as well as the aging period after gelation, are the key points that strongly influence the emulsion gels properties. The emulsion gels can be classified into three categories based on biopolymers composition in a gel matrix, including the following: (i) protein-based emulsion gels (e.g., caseins, gelatin, soy, and whey proteins), (ii) polysaccharide-based emulsion gels (e.g., alginate, starch, pectin, and xanthan gum), and (iii) mixed emulsion gels (e.g., soy protein isolate-beet pectin, xanthan gum-guar gum, and zein-sodium caseinate-propylene glycol alginate). In recent years, researchers have mostly focused on the formation of protein-based emulsion gels, which might be due to their excellent emulsifying properties, relatively easy processing (gelation), and inheritable nutritional composition (19, 36).

## Protein-Based Emulsion Gels

Protein emulsion gels usually had a relatively high protein concentration in the gel matrix, and caseins, gelatin, soy, and whey proteins are the widely used biopolymers in emulsion gels production due to their abundance, renewable resources, and promising emulsifying and gelling properties. Gelation techniques including heat-set (heating), and cold-set such as acidification (acid-induced), ethanol-induced, enzyme treatment, salt addition (salt-induced), and hydrostatic pressure-induced approaches have been applied to synthesize the protein-based emulsion gels (12, 37).

Recently, Luo et al. (19) synthesized whey protein emulsion gels and then microgel particles with an average size of 0.5 ± 0.05 μm *via* heating (90°C for 20 min) followed by cooling (4°C for overnight) method to enhance the bioaccessibility of capsaicinoids. The optimized formula includes whey protein (10 wt%), soybean oil (19.98 wt%), and capsaicinoids (0.02 wt%) used in the preparation of emulsion gels. The *in vitro* study results suggested that emulsion gels as a delivery system significantly increased the bioaccessibility of encapsulated capsaicinoids and showed a positive correlation with the extent of lipid digestion. The bioaccessibility indicated the release of capsaicinoids from gel matrix during digestion and their solubilization in the aqueous phase in the gastrointestinal tract. Fu et al. (36) fabricated whey protein emulsion gels containing medium chain triglyceride and cinnamaldehyde oils by the heating method. The scanning electron microscopy results showed that cross-linking occurred between the whey proteins and cinnamaldehyde at the oil-water interfaces leading to an effective reduction in viscosity, an increase in viscoelasticity, and smaller and uniform pore size in emulsion gels. The *in vitro* study on gastrointestinal fate showed that protein-based gel had slower disintegration than protein-cinnamaldehyde gel and revealed a faster disintegration due to the addition of cinnamaldehyde that softened the gel.

Lv et al. (38) produced Pickering emulsion gels containing canola oil stabilized by whey protein isolate gelled particles aimed to encapsulate curcumin. The formed gels had the highest loading efficiency of 90.3%, which contributed to the gel's compact structure (solid-like) that retained the maximum percentage of curcumin. The *in vitro* release under the gastric and intestinal conditions revealed that emulsion gel had a slower release rate than liquid emulsion ascribed to the gel-like structure which showed a better resistance against the hydrolysis by pepsin. Moreover, the emulsion gel encapsulating curcumin hindered the degradation and showed remarkable stability, i.e., >70% amount remained compared to only 7% of control (without any protection) during storage of 240 min under the light. Tan and his team prepared highly concentrated emulsion gels as nutraceuticals cargos by encapsulating 80% sunflower oil by employing gelatin particles (~200 nm) as the emulsifiers at concentrations of 0.3–1.5 wt%. The formed gels remained stable even after 90 days of storage and embedded β-carotene showed a very high retention rate (90%) than that of bulk oil (8%) recorded after 27-days (39, 40). Xu et al. (41) produced emulsion gels containing 80% dodecane stabilized by β-conglycinin Pickering particles (0.2–1 wt%), and formed gels showed excellent stability during 60-days storage as well as heating at 100°C for 15 min. Furthermore, a progressive decrease was observed in the size of the droplets from 60 to 24 μm with an increase in particle concentration from 0.2 to 1 wt%. Thus, emulsion gels and Pickering emulsion gels can improve the digestion process along with providing protection to the embedded lipophilic compounds attributed to the compact gel structure.

## Polysaccharide-Based Emulsion Gels

These emulsion gels comprise polysaccharide food polymers with the gelation capacity that depends on their source and structure, and alginate, agarose, modified starches (e.g., octenyl succinic anhydride), carrageenan, curdlan, inulin, konjac gum, and xanthan gum were the commonly employed biopolymers in the formation of emulsion gels. Various methods, including heating, heating and cooling, high shear, freeze-thaw cycles, pH, ions induced (Ca^2+^ and K^+^), and salts addition (CaCl_2_) can be applied to induce gelation leading to the formation of emulsion gels (1, 37). Polysaccharide-based emulsion gels are thermoreversible and their derived colloidal particles are also considered less efficient emulsifiers to produce emulsion gels compared to protein-based emulsion gels that exhibit long-term stability because of protein molecules that not only act as gel substrates but are excellent stabilizers (1, 10, 12, 28). However, polysaccharide-based biopolymers are more effective to increase viscosity and also exhibit inherent resistance to digestive enzymes, such attributes make them particularly suitable as delivery vehicles for bioactive compounds that require a prolonged digestive journey for the encapsulates (20, 42, 43).

Torres et al. (30) fabricated starch-based emulsion gels through heat-induced gelation, containing sunflower oil volume fraction (5–20 wt%), wheat starch (15–20 wt%), and octenyl succinic anhydride (OSA) https://www.sciencedirect.com/topics/biochemistry-genetics-and-molecular-biology/modified-starch modified starch (0.5–2 wt%). The results indicated that when starch was used at a concentration of 20 wt% and oil at 5–15 wt%, the gel elastic modulus increased by 50%, whereas a further increase in oil content (20 wt%) strengthened the gel with an increment in the elastic modulus up to 70%. This reinforcement in the gel matrix is attributed to the hydrophobic interactions between oil droplets and interfacial starch, and hydrogen bonding among starch polymers specifically amylose molecules to form 3D networks. The authors also produced gel particles with a diameter ranging from 5 to 50 μm *via* a top-down shearing of the formed gels and suggested that these novel emulsion gels and derived microgel particles can be employed in the delivery of bioactive substances in various food and personal care products.

Mokhtari et al. (44) developed alginate nanogels *via* emulsification and internal gelation induced by https://www.sciencedirect.com/topics/chemistry/calcium-chloride calcium chloride to deliver nutraceuticals with high encapsulation efficiency. The results showed that sodium alginate (0.5%), canola oil (400 ml), calcium chloride (0.05 M), and Tween 80 (100 ml) were the optimized concentrations in formulating alginate-based gel nanoparticles. Moreover, the nanocarriers derived from gel showed a spherical shape and a higher encapsulation yield was obtained with the increasing alginate amount due to increased viscosity that imparted more cohesion property leading to high entrapment efficiency. Furthermore, the gel particle size and encapsulation yield were found highly proportional to the alginate concentrations as high amounts lead to higher while small amounts resulted in lower values. Zhang et al. (42) prepared emulsion gel using carrageenan as gel matrix stabilized by mixed colloidal Pickering nanoparticles made of zein-carboxymethyl dextrin biopolymers and *N*-ethyl-N-(3-dimethylaminopropyl) carbodiimide as the crosslinking agent. The *in vitro* digestion analysis suggested that the bioaccessibility of curcumin in crosslinked emulsion gel was decreased compared to emulsion due to the spatial networks developed in emulsion gel. Briefly, the network structure slowed down the digestive enzymes' diffusion into the gel matrix, thus delaying the https://www.sciencedirect.com/topics/biochemistry-genetics-and-molecular-biology/enzymatic-hydrolysis hydrolysis and digestion of oil droplets encapsulating curcumin and subsequently decreasing the bioaccessibility. In addition, this study's investigations revealed that photochemical and thermal stability of the impregnated curcumin in emulsion gel was significantly improved due to crosslinking and exhibited high retention rates of 90.7 and 82.2% under light and heat, respectively.

## Mixed Emulsion Gels

Interest in the production of mixed gels has attracted more attention from researchers since they offer enriched gelling behaviors and more precise control on the physicochemical, rheological, and functional properties over the individual emulsion gels. Le et al. (20) reported that mixed gels (protein and polysaccharide) had outstanding water-holding properties (up to 600 g/g of mixed gel), and were also capable to produce a diverse range of microstructures that can be further exploited to bring desirable textures and sensory attributes. For instance, during the process of homogenization, the polysaccharide part imparts better stability against environmental conditions such as pH, temperature, and ionic strength, while the protein part contributes to producing fine droplets size through excellent emulsifying capacity leading to a homogenous structure (10). Gelation methods, including heating, cooling after heating, high shear, enzyme treatment, and coacervation can be applied to produce mixed emulsion gels (20, 45).

Particularly, coacervation is a commonly employed method in the formation of mixed gels from oppositely charged biopolymers, (e.g., protein-protein, polysaccharide-polysaccharide, and protein-polysaccharide), by regulating the mixture ratio (biopolymer type and concentration), temperature, pH, ionic strength, *etc*. During this process, the charged species (e.g., H^+^ and OH^−^) adsorbed on biopolymers surfaces interact through electrostatic complexation and develop 3D networks leading to gel formation (1, 20). Table 1 describes different mixed emulsion gels combinations that include the following: (i) protein-protein (72), (ii) polysaccharide-polysaccharide (43), and (iii) protein-polysaccharide, etc. (68).

**Table 1:** Gel-based functional delivery systems, including protein-, polysaccharide-, and mixed emulsion gels (emulgels).

**Delivery systems**	**Gel matrix**	**Oil phase**	**Bioactives**	**Applications**	**References**
**Protein-based emulsion gels**	Whey protein isolate	Corn oil	Probiotics	Encapsulation and controlled release	Gao et al. (29)
	Soy protein isolate	Olive oil (40%)	Polyphenols	Encapsulation and controlled release	Munoz-Gonzalez et al. (46)
	Soy protein	Soybean oil (50%)	Inulin	Fat replacement	de Souza Paglarini et al. (33)
	Whey protein isolate	Soybean oil (50%, v/v)	…….	Functional food	Xi et al. (47)
	Whey protein isolat	Mixed oils (coconut & corn, 20% of emulsion)	β-carotene	Encapsulation and controlled release	Lu et al. (48)
	Whey protein isolat	Soybean oil (19.98 wt%)	Capsaicinoids	Encapsulation and controlled release	Luo et al. (49)
	Whey protein isolate	Soybean oil (30%)	…….	Encapsulation and controlled release	Mantovani et al. (50)
	Whey protein isolate	Soybean oil (30%)	Retinol (vit. A)	Encapsulation and controlled release	Beaulieu et al. (51)
	β-lactoglobulin	Sunflower oil (30%)	α-Tocopherol (vit. E)	Encapsulation and controlled release	Liang et al. (52)
	Soy protein isolate	…….	Riboflavin (vit. B2)	Encapsulation and controlled release	Maltais et al. (21)
	Wheat gluten	Corn oil (56%)	EGCG + quercetin	Encapsulation and controlled release	Chen et al. (53)
**Polysaccharide-based emulsion gels**	Carrageenan	Soybean oil (50%)	..….	Fat replacement	Paglarini et al. (28)
	Alginate	Canola oil (40, 60, and 80%)	Peppermint extract	Encapsulation and controlled release	Mokhtari et al. (44)
	Gellan gum	Soybean oil (60%)	Probiotics	Probiotics delivery	Picone et al. (54)
	Starch	Soy oil (85%)	..….	Texture design and modifications	Yang et al. (55)
	Rice starch	Sunflower oil (40%)	……	Texture design and modifications	Zhang et al. (56)
	Sodium alginate	Paraffin oil (0.2%)	Probiotics	Probiotics delivery	Qi et al. (57)
	Sodium alginate	Tea seed oil (0.2 g)	Curcumin	Encapsulation and controlled release	Xu et al. (58)
**Mixed emulsion gels**	Whey protein isolate-soy protein isolate	Sodium alginate (0.4%	..….	Texture design and modifications	Lin et al. (11)
Protein-protein					
	Whey protein- lactoferrin	Corn oil (30 g)	..….	Reduced-fat products	Yan et al. (59)
	Whey protein-soy protein	Olive oil, linseed oil, and fish oil (44.39, 37.87, and 17.74%	Fatty acids (n-3) and condensed tannins	Encapsulation and controlled release	Freire et al. (60)
Polysaccharide-polysaccharide	Alginate-konjac glucomannan	Rapeseed oil (5–30%)	…...	Fat replacement	Yang et al. (61)
	Gellan gum- Pectin-carrageenan-xanthan Gum	Corn oil (10%)	Quercetin	Encapsulation and controlled release	Chen et al. (53)
	Xanthan gum-guar gum	Sunflower oil (41%)	Probiotics	Probiotics delivery	Pandey et al. (43)
Protein-polysaccharide	whey protein isolate-carrageenan	MCT oil (4 mL)	Curcumin	Encapsulation and controlled release	Su et al. (62)
	black soybean protein-sodium alginate	Soybean oil	Insulin and quercetin	Encapsulation and controlled release	Han et al. (63)
	Whey protein isolate-sodium alginate	Corn oil (20% v/v)	Lycopene	Encapsulation and controlled release	Liu et al. (64)
	Soy proteinisolate-pectin	Soybean oil [6% (v/v)]	β-carotene	Encapsulation and controlled release	Zhang et al. (65)
	Whey protein isolate-alginate	Sunflower oil (0.5–20%)	α-Tocopherol + resveratrol	Encapsulation and controlled release	Feng et al. (66)
	Whey protein isolate-xanthan gum	Babacu oil and tristearin (4%)	Curcumin	Fat replacement	Geremias-Andrade et al. (67)
	Soy protein-sugar beet pectin	Corn oil (15%)	Ethyl butyrate	Encapsulation and controlled release	Hou et al. (68)
	Whey protein isolate- rice starch	Corn oil (2–8%)	Carotenoids	Encapsulation and controlled release	Mun et al. (69)
Polysaccharide-emulsifier	Kappa-carrageenan-polysorbate 80	Algae oil	Catechins	Encapsulation and controlled release	Alejandre et al. (70)
Protein-protein-polysaccharide	Zein- sodium caseinate-propylene glycol alginate	Soybean oil (80%)	……..	Texture design and modification	Sun et al. (71)

Pandey et al. (43) formulated mixed emulsion gel using sunflower oil and a combination of xanthan gum and guar gum for the delivery of https://www.sciencedirect.com/topics/biochemistry-genetics-and-molecular-biology/lactobacillus-plantarum *Lactobacillus plantarum* 299v. The mechanical strength and disintegration investigations on formed gel showed significant improvements in the mechanical stability and gastric acid resistance due to the combination of the xanthan gum and guar gum in the dispersed phase of the formulation. Moreover, the natural gums-based emulsion gel stored at different conditions including 4, −20, and −196°C, revealed higher survival rates of encapsulated probiotics in the emulsion gels compared to control. Hou et al. (68) designed mixed emulsion gels *via* the enzymatic (mTGase) gelation method, comprising flavored corn oil and stabilized by soy protein isolate-sugar beet pectin complexes. Briefly, complex emulsified emulsion gels presented more compact structures due to the formation of strong interfacial networks ascribed to higher emulsifier absorption at the oil-water interfaces. In addition, gas chromatography analysis revealed that the ethyl butyrate release rate was significantly lower before and after the mastication process in emulsion gels due to their compact structure.

Zou et al. (73) prepared Pickering emulsion gels containing corn oil volume fraction of 50% stabilized by complex Pickering particles (zein-tannic acid- 1-1.5 wt%) having a three-phase contact angle value of ~86° and high interfacial activity. The formed emulsion gels stabilized by complex Pickering particles showed homogeneous structure and good stability over 30-days of storage without any signs of oiling-off (creaming and phase separation) due to their transformation from a liquid state to a semisolid state ascribed to particles networks. Wei et al. (72) produced highly concentrated emulsion gels using ovotransferrin-lysozyme complex Pickering particles as the stabilizers, and by increasing particle concentration from 0.5 to 2 wt% the size of the droplets decreased from 81.4 to 42.4 μm at a fixed oil phase (75%). The formed emulsion gels presented excellent stability during long-term storage and inhibited the phase separation phenomenon because of particles networks ascribed to electrostatic interactions. The Pickering particles stabilized emulsion gels enhanced the bioaccessibility of the impregnated curcumin by 22.2%, indicating gels as an effective delivery system for lipophilic bioactive substances. (74) fabricated Pickering emulsion gels by encapsulating medium chain triglyceride oil (50-60%) stabilized by complex colloidal particles (zein-pullulan - 2 wt%). The resultant gels showed excellent stability against coalescence and phase separation and no oil leakage was noticed even after 30-days of storage at room temperature. The stability of emulsion gels was due to the formation of compact interfacial layers by the colloidal particles around the oil droplets, indicating their potential as a delivery system for bioactive substances to design better food formulations.

## Gel-Body Interactions

### Texture Perception During Oral Processing

Texture perception is a complex process that correlates with the properties of food such as chemical composition and structure as well as physiochemical, mechanical, and enzymatic changes that occur during oral processing (75, 76). Briefly, texture indicates the rheology (hardness and brittleness-chewing) and tribology (creaminess and smoothness-lubrication) related food attributes sensed at early and later stages of oral processing, respectively (1, 77–79). It has been found that the rheological properties were correlated with the fracture stress and strain and determined by the hardness and brittleness of food, whereas tribological properties were linked with the friction coefficient perceived by creaminess or smoothness (76, 80, 81). The rheological and tribological properties of gels can be finely tuned by the gel composition (biopolymer type and concentration), structure, and mesh size to obtain the desired sensory attributes.

In recent years, interest in the application of emulsion gels as a potential tool to mimic the food texture without affecting their sensory perception by designing gels with reduced fat, sugar, and salt has rapidly increased. The incorporation of emulsion gel or derived particles into semisolid food formulations could mimic the perception of fat because of the significant reduction in friction coefficient through ball-bearing in the case of whey proteins (82, 83) and enzymatic reactions for starch particles (84). Interestingly, mouth-melting gels (e.g., gelatin-based gels) had excellent potential to increase the surface lubrication of foods to achieve the desired mouthfeel of fat perception (85). For instance, gelatin-based gels remain in a gel state at room temperature but start melting at mouth temperature (37°C), thus could be effectively used in many food formulations to mimic fat perception. In addition, emulsion gels can also avoid undesirable sensory attributes of drugs or bioactive compounds by separating them from the taste bud receptors in the mouth and delaying their release in the digestive tract (26, 27).

Zhang et al. (56) used enzymatically hydrolyzed rice starch (20 g) and sunflower oil (40 g) to fabricate emulsion-filled gels, and the resulting gels showed a comparable texture including hardness and cohesiveness similar to fat. Moreover, the concentration of starch in the formulation directly influenced the textural properties and flow behavior of formed gels. Briefly, the firmness of emulsion gels was strengthened with the increase in the concentration of starch, since the high content of amylose and longer chains of amylopectin led to the formation of harder emulsion gels. Luo et al. (49) prepared whey protein-based emulsion gels comprising whey protein isolate 895 (10 wt%), soybean oil (19.98 wt%), and capsaicinoids (0.02 wt%), and evaluated their oral breakdown behavior and mouth burn perception. The capsaicinoids-loaded whey protein emulsion gels revealed a lower mouth burn perception due to higher mechanical strength that resulted in a slower diffusion rate of the encapsulates from the gel matrix and subsequently a lower mouth burn. These results suggested that emulsion gels had promising textural potentials which could be utilized to mimic desirable sensorial perceptions as well as for masking the undesirable flavors of bioactive compounds in food formulations while maintaining their consumer acceptability.

### Digestion

The digestion kinetics of food-grade polymer-based gels are mainly influenced by the gelation process, for instance, gelation (acid and heat) of milk proteins slowed down their digestion rate and prolonged the gastric retention time without affecting the enzymatic cleavage sites (86, 87). Likewise, the majority of polysaccharides (dietary fiber form) in the gel state can significantly increase the gastric emptying time due to the thickening effect that becomes prominent upon their transformation from polymer to gel particles (88). Moreover, gelation also delays the diffusion of digestive enzymes into the gel matrix, thus digestion process of bioactive compounds can be substantially delayed by encapsulating into food gels (42). Therefore, *via* decreasing the digestion rate and prolonging gastric retention time, gels impart health-promoting effects, including enhanced satiety to attenuate obesity, good control of glucose and cholesterol metabolism to prevent chronic disorders, etc. (26, 88, 89).

The gels digestion process leads to the release of the encapsulated bioactive compounds that takes place through different mechanisms, including disintegration, swelling, molecular interactions, and erosion (1, 17, 18). In the process of gel erosion, the human enzymes work synergistically with the pH responses (e.g., oral cavity, stomach, small intestine, and colon) to initiate the controlled release of embedded bioactive agents at the target sites such as the small intestine and colon. The pH differences of the gastrointestinal tract bring changes to the overall charge on the surface of biopolymers, thus, charge shifting and different digestion behaviors in combination with the interior environment make gels promising vehicles for the efficient delivery of sensitive bioactive substances. For small intestine delivery, a protein can be entrapped in calcium alginate gel particles at pH 3 due to the opposite charges on protein and alginate biopolymers, and protein can be easily released in the small intestine at pH 7 because at this pH both biopolymers possess negative charges (90). Therefore, the enzymes diversity and pH responses show the potential to develop functional delivery systems for the encapsulation, protection, and release of bioactive at targeted sites, that could be further fine-tuned by manipulating the differences in the ionic strengths between the food gels formulations and the gastrointestinal environment (17, 91, 92).

Lin et al. (11) fabricated alginate-based emulsion gels stabilized by proteins (whey protein and soy protein isolates as emulsifiers) and tomato-derived lycopene was encapsulated in gels to study the *in vitro* digestion and its release behavior. The results showed a delayed release of the encapsulated lycopene from emulsion gels; whey protein isolate stabilized alginate emulsion gel release commenced at 4-4.5 h, soy protein isolate stabilized gel at 3.5-4 h, whereas an early release of 2.5-3 h was observed in alginate-based gel without any protein. The release corresponds to the start point of the structural collapse and degradation of gel networks after swelling during *in vitro* digestion of the emulsion gels. The whey protein had a better emulsifying capacity and also developed stronger interactions with alginate, thus improving the gel properties such as increasing Young's modulus of emulsion gel. A higher Young's modulus could retard the swelling process and subsequently prevents the collapse of gel structure during the *in vitro* gastric digestion. Thus, controlled release of encapsulated lycopene was achieved *via* delayed degradation of gel matrix during the *in vitro* intestinal digestion. Therefore, emulsion gels can be employed to protect the health-promoting substances from the harsh conditions of the gastrointestinal tract and deliver them to specific target sites with improved digestion and bioaccessibility (93, 94).

### Absorption

It is a proven fact that undigested food is difficult to transport leading to poor absorption of the inheritable nutrients into the body because of barriers in the gastrointestinal tract epithelial cells and especially the mucous layer. The mucous layer comprises a 3D network of mucin glycosylated protein fibers with an average mesh size of ~100 nm (45, 95, 96). In this regard, emulsion gels and derived particles with optimum surface attributes (e.g., size, shape, surface charge, and hydrophilicity/hydrophobicity) had the potential to enhance the mucosal permeability and maximize the bioavailability of embedded bioactive compounds (37, 94). Among the biopolymers, chitosan exhibits the potential to adhere and travel through the mucous layers to improve cellular uptake *via* reversible depolymerization of cellular actin and strong interactions with the protein molecules. Thus, chitosan emulsion gels are popular delivery cargos with the superiority of enhancing cellular permeability without damaging epithelial cells (93, 96). For particles traversing through the epithelial cells, the main pathways include paracellular migration and transcellular translocation through tight junctions and enterocytes or M cells, respectively. Therefore, increasing the translocation of loaded gel particles is an important strategy for efficient delivery of the biomolecules with low mucosal permeability, including proteins, peptides, antimicrobial agents, nutraceuticals, and functional food ingredients (37, 93, 94, 97).

Haug et al. (98) designed gelatin-based emulsion gels and investigated their capability as delivery vehicles to boost the bioavailability of omega-3 fatty acids including eicosapentaenoic acid (EPA) and docosahexaenoic acid (DHA). The study's findings revealed that emulsion gels significantly increased the EPA and EPA + DHA levels in the blood plasma by 44.9 and 43.3%, respectively, as compared to gelatin capsules oral intake. This significant increase was attributed to the pre-emulsification of the fish oil as well as the design of the delivery vehicles (chewable and soft (emulsion gels) vs. intact and solid (capsules). Compared to gel capsules consumption, EPA and EPA + DHA loaded emulsion gels showed the highest increase of 100.4 and 105.6%, respectively, indicating gels' potential as efficient delivery systems in improving the bioavailability of gel matrix embedded functional ingredients.

### Food Applications

Emulsion gels and derived particulates have emerged as promising delivery systems for industrial food applications due to their unique properties such as protection of functional food additives, controlled release of bioactive substances, and improved digestion and absorption of macro- and micronutrients in the gastrointestinal tract (37, 75, 99, 100). The industrial food applications of emulsion gels, including encapsulation and controlled release, texture design and modification, fat replacement, and probiotics delivery with enhanced viability are discussed in the following section.

### Encapsulation and Controlled Release

Encapsulation is a promising technique widely employed for the protection and targeted delivery of bioactive compounds due to the superior stability of embedded substances against chemical, physical, and environmental stresses, and their desired controlled release (e.g., fast or sustained). To date, different delivery systems have been successfully developed with desirable structures and characteristics such as protected encapsulation and delivery of various bioactive nutrients with improved health benefits (17, 37, 101). In recent years, emulsion gels have garnered considerable attention as promising encapsulation and delivery cargos due to their superior properties for many food applications.

**Figure 4:**
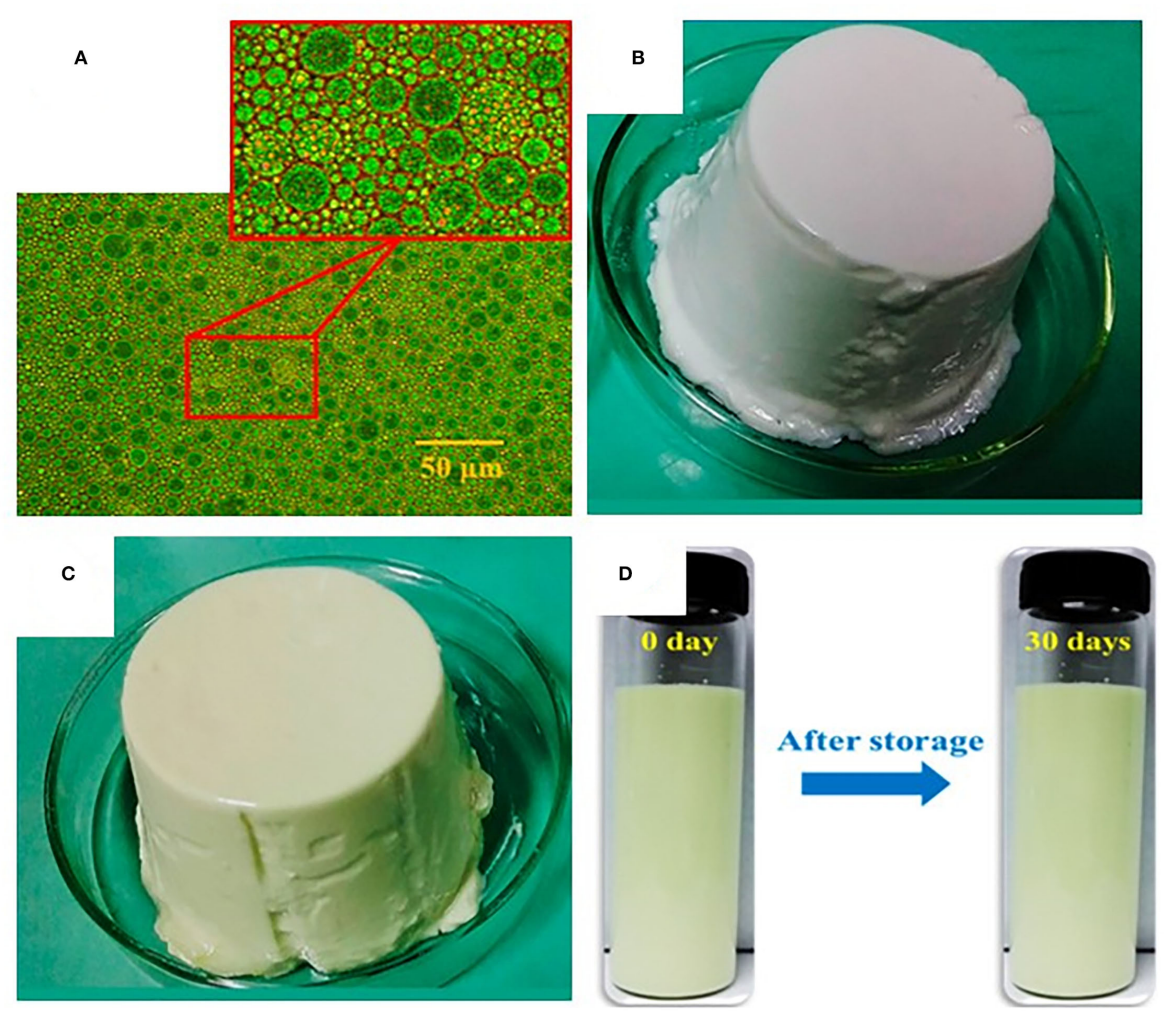
Optical microscopic image and visual appearances of emulsion gels; **(A)** Emulsion dispersion droplets, **(B)** Blank emulsion gel, and **(C)** Epigallocatechin-3-gallate and quercetin co-loaded emulsion gel. The formed gel showed an encapsulation efficiency of 65.5 and 97.2%, whereas enhanced the bioaccessibility by 48.4 and 49% for (-)-epigallocatechin-3-gallate and quercetin, respectively. In addition, emulsion gel showed lower release rates of 73.3 and 31.7% and improved stability by 63.6 and 82.3% for epigallocatechin-3-gallate and quercetin after 8-h incubation in specific environmental conditions (simulated intestinal fluid) and remained stable to phase separation during 30-days storage at 4°C **(D)** (103).

Xu et al. (41) prepared alginate-based nanoemulsion-filled gels fabricated by a facial approach of self-emulsification and sodium alginate ionic gelation. The formed gel loaded with curcumin showed an average particle size of 0.46 ± 0.02 mm, a loading capacity of 7.25 ± 3.16 mg/g, and encapsulation efficiency of 99.15 ± 0.85%, and the release rate was found significantly higher at pH 9 than at pH 7. The results showed that the alkaline condition (pH 9) achieved the half-release time of curcumin (50% release) in just 3 h due to an accelerated corrosion process compared to the neutral environment (pH 7) that provided stability to the curcumin loaded emulsion filled gels. Zhang et al. (102) produced gel as a delivery system from whey protein isolate through heat gelation (24 h at 85°C) for the encapsulation of β-carotene. The encapsulation efficiency was greatly improved from 76.55 to 92.11% than that of untreated whey protein isolate. The layers of protein isolate enhanced the protection of β-carotene, resulting in improved digestion resistance and subsequently increased bioavailability. Chen et al. (103) produced Pickering particle-stabilized emulsion gels *via* emulsification and pH adjustment and coencapsulated (-)-epigallocatechin-3-gallate (EGCG-hydrophilic bioactive) in the inner water phase, whereas quercetin (lipophilic bioactive) in the oil phase (Figure 4). The formed gels used to coencapsulate bioactives showed an encapsulation efficiency of 65.5 and 97.2% for (-)-epigallocatechin-3-gallate and quercetin in the aqueous phase and oil phase, respectively. Furthermore, *in vitro* study revealed that gels improved the bioaccessibility by 48.4 and 49% compared to control (water suspension) by 25.8 and 15%, for epigallocatechin-3-gallate and quercetin, respectively. Thus, emulsion gels as delivery systems had the potential for the encapsulation and coencapsulation of hydrophilic and lipophilic bioactive along with high encapsulation efficiency and controlled release features to enhance their bioaccessibility. In summary, gels are efficient cargos that could be used in improving the digestion, bioaccessibility, and bioavailability of bioactive compounds, functional ingredients, and pharmaceuticals.

### Texture Design and Modification

The increasing consumers' awareness and industrial demands for clean-label food products have challenged the use of traditional synthetic food additives (e.g., thickeners, moisture absorbers, and emulsifiers) in designing food structures with desired properties. Employing emulsion gels is appropriate for various industrial food applications, for instance, they can be employed for modifying the texture and designing safer and healthier functional foods with improved physicochemical properties. Furthermore, the texture of emulsion gels can be fabricated by varying the biopolymer type and concentration, Pickering particles concentration (emulsifier), addition of functional additives, and processing conditions such as pH, ionic strength, and temperature (8, 12).

Zhang et al. (56) prepared emulsion-filled gels (EFGs) using enzymatically hydrolyzed rice starches (ERS) instead of native rice starch. The amount of starch in the gel medium had a direct impact on the final properties of ERS-EFGs. The addition of starch was found responsible for increasing the storage modulus (G′) and loss modulus (G″), as well as improving the firmness and freeze-thaw stability of resultant ERS-EFGs. On the other hand, the higher amount of emulsion droplets diluted the starch concentration, causing a reduction in both G′ and G″, freeze-thaw stability, and firmness. Taktak et al. (8) synthesized the gelatin-based emulsion gels from European eel skin gelatin (ESG) and European oil (EO). The emulsion gels were prepared using the weight of EO:ESG in 1:2 and 1:4 (w/w) ratio through the homogenization process or homogenization followed by sonication. The textural properties such as hardness (7.87 N), masticability (35.94 Nmm), breaking strength (4.72 N), and rigidity (1.29 N/mm) were higher in gelatin gel (control) compared to gelatin based emulsion gels, indicating that emulsion gels were more flexible than gelatin gel. Gao et al. (29) synthesized high internal phase emulsion gels, prepared by whey protein isolate and pectin, and gelled by the addition of D-(+)-gluconic acid δ-lactone and calcium to form double networks high internal phase emulsion gels. The structural properties, such as hardness (220.77 ± 11.07 g), adhesiveness (168.4 ± 31.18 g/s), gumminess (129.98 ± 4.63 g), and chewiness (108.41 ± 23.91 g) of the internal phase emulsion gel (whey protein isolate and 2% pectin), were significantly higher as compared to hardness (125.59 ± 18.64 g), adhesiveness (35.2 ± 8.1 g/s), gumminess (51.02 ± 16.85 g), and chewiness (50.79 ± 17.04 g) of a hydrogel (prepared with similar composition as in internal phase emulsion gel). Thus, the incorporation of emulsion gels in foods as functional colloids exhibits the potential to regulate textural (rheology and tribology) and functional properties such as reduced sugar, salt, or cholesterol contents.

### Fat Replacement

Worldwide, cardiovascular diseases developed due to excessive intake of trans-fats are considered a major cause of morbidity and mortality (104). Mozaffarian et al. (105) reported that consumption of processed foods rich in saturated and artificial trans-fats elevated the prevalence of coronary heart disease from 23 to 29% when energy intake was increased by only 2% from foods containing trans-fats. Moreover, the United States Food and Drug Administration had also put a ban on partially hydrogenated oils used in processed foods, since they are a major source of trans-fats (106). Thus, the consumers' concerns about the harmful effects of fat consumption and the recent policy of the FDA related to the exclusion of trans-fat from food products have together attracted increased attention for needed innovation (107). In this regard, emulsion-gel technology may be employed to develop food products without trans fats and also to transform partially hydrogenated oils into semi-solid forms such as viscoelastic gels with zero trans fats or less saturated fats as a substitute for solid fats.

Nacak et al. (34) prepared emulsion gel comprising oil phase [peanut oil:linseed oil (10:1) and polyglycerol polyricinoleate (3.2 g)], and aqueous phase [water (37 g), inulin (8 g), egg white powder (3 g), and gelatin (2 g)], per 100 g of emulsion by heating at 55°C in water bath followed by emulsification at 700 rpm for 3 min. The formed gel was utilized to replace the beef fat partially/completely (50 and 100%) in sausages and found a 40% reduction in total fat content and 27% in case of energy content. Interestingly, the content of total saturated fatty acids (21.46 ± 0.4) and cholesterol (27.32 ± 0.6) was successfully decreased, while obvious boosts were seen in mono-unsaturated fatty acids (45.95 ± 0.14) and poly-unsaturated fatty acids (29.78 ± .22) in sausages containing emulsion gels (100% beef fat replacement) as compared to control. Likewise, de Souza Paglarini et al. (33) produced emulsion gels by encapsulating 50% soybean oil in the soy protein isolate (4%) and inulin (16.5%) as gel substrate to replace animal fat. A decline in fat (11 to 34%) was calculated in the reformulated products, furthermore, the least total fat content (190.4 ± 6.3), higher fiber content (2.97 %), and high amount of polyunsaturated fatty acid (80.37 ± 3.72) observed in emulsion gels incorporated sausages. Notably, the incorporation of emulsion gel replacing animal fat also offered better sensory properties such as texture, flavor, aroma, and overall liking. These findings indicated that emulsion gels had potential as animal fat substitutes and could be used in formulating healthier food products with a better fatty acids composition and sensory score. Liu et al. (108) used wheat gluten protein particles (1 wt%) as Pickering emulsifiers and prepared emulsion gels by encapsulating sunflower oil as a mayonnaise substitute. The formed gels presented excellent thermal stability at 90°C for 30 min than the mayonnaise, which showed a complete collapse with oil leakage. These findings suggested that emulsion gels have had better nutritional ratios, healthier lipid composition, and acceptable sensory features which could be used to replace animal fat and as a safe alternative to partially hydrogenated oils.

### Probiotics Delivery

Probiotics are the viable microorganisms in the human gastrointestinal tract that impart health-promoting characteristics by regulating the balance of gut microflora (e.g., *Bifidobacterium* and *Lactobacillus*) (109, 110). For example, probiotics present in the intestinal tract exert multiple health benefits e.g., improved gastrointestinal tract health, enhanced immunity, reduced bad-cholesterol levels, and harmful microorganisms growth inhibition (111, 112). However, environmental and processing conditions such as high relative humidity and high temperatures greatly affect probiotics viability (113). Recently, biopolymer-based emulsion gels have emerged as a promising delivery system for probiotics targeted delivery with enhanced viability by protecting them from harsh environmental conditions during processing and digestion.

Gao et al. (29) developed high internal phase emulsion gels from whey protein isolate and pectin biopolymers to encapsulate and deliver *Bifidobacterium lactis*. The results showed that the viability of encapsulated probiotics was significantly higher in high internal phase emulsion gel (5.31 log CFU/ml) than hydrogel (4.66 5.31 log CFU/ml) after a heat treatment at 65°C for 30 min ascribed to the fact that gel pores filled with oil droplets effectively protected and minimized the effect of heating on probiotics. In addition, the strength, shear viscosity, water holding capacity, and stability of gels increased with an increase in the concentration of pectin from 0 to 2%. Qi et al. (57) developed smooth and spherical shaped micro-beads by emulsion-gelation method with sizes ranging from 300 to 500 μm as a delivery system for *Saccharomyces boulardii* and *Enterococcus faecium*. The formed gels showed that *S. boulardii* and *E. faecium* grew well and their survival rate improved by 25 and 40%, respectively, compared to controls under high temperature and high humidity. The survival rate in gastric juice for *S. boulardii* (60%) was significantly higher than *E. faecium* (25%), but in the case of intestinal juice, a higher rate was noticed for *E. faecium* (20%) than *S. boulardii* (15%). Picone et al. (54) synthesized gellan gum-based spherical microbeads with a diameter of 1.85 μm *via* the emulsion-gelation method to deliver *Lactobacillus rhamnosus* with a higher survival rate. The formed emulsion gel showed the encapsulated probiotics viability as 77% which was significantly higher than the non-gelled emulsion of 66%. The *in vitro* study findings suggested that emulsion-gelation improved the resistance of the embedded probiotics which remained stable to oral and gastric digestions. Based on the aforementioned findings, emulsion gels have emerged as model probiotics delivery systems that not only protected the encapsulated probiotics from adverse *in vitro* and *in vivo* conditions but also ensured their targeted delivery with enhanced viability. In addition, biopolymers as wall material also provide nutrients to probiotics and the host due to biopolymers' inheritable nutrient profiles, suggesting their potential to deliver probiotics in the gastrointestinal tract and development of functional formulations such as fermented foods.

## Conclusions and Future Trends

**Figure 5:**
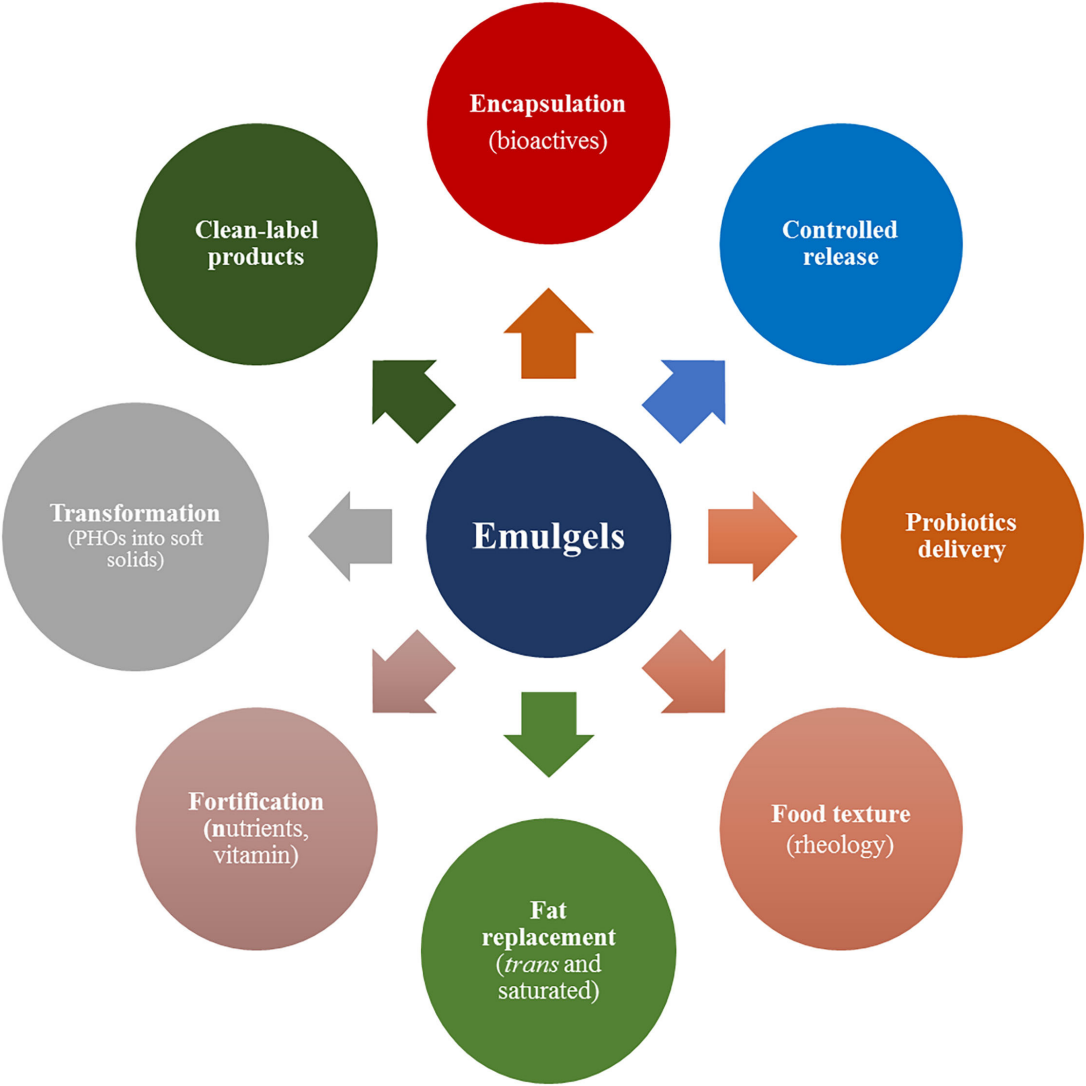
Emerging industrial food applications of emulsion gels (emulgels).

Emulsion gel is an emulsion dispersion-filled gel matrix, wherein at least one phase either continuous phase or dispersed phase of emulsion creates 3D networks leading to the formation of semisolid texture. In this review, the authors have discussed the classification of emulsion gels based on their constitutive nature of the polymers (i.e., proteins, polysaccharides, and mixed emulsion gels), and gel-body interactions including sensorial textures, digestion, and absorption. The tunable attractive properties (morphology, mechanical, and functional) and unique characteristics (biocompatible, biodegradable, eco-friendly, and cost-competitive sustainable biomaterials) make emulsion gels promising functional colloids and delivery vehicles for different food applications (encapsulation, controlled release, texture design, fat replacement, probiotics delivery, designer foods, and so on). Based on their properties and characteristics emulsion gels could be effectively utilized in the processing, transportation, and targeted delivery of food additives, nutraceuticals, probiotics, and functional ingredients such as flavors, natural pigments, minerals, and vitamins (Figure 5 and Table 1). In addition, emulsion gels can tailor breakdown behavior and sensory perception of food, protect the bioactive substances against adverse conditions, modify their dispersible status in the food matrixes, control their release time and rate, and eventually enhance their bioavailability.

Emulsion gels could possibly be used to develop innovative stimuli-responsive gels which may alter their morphology and properties upon exposure to any external stimuli such as enzymes, light, temperature, pressure, and pathogenic microorganisms. Moreover, a combination of stimuli-responsive gels with biological entities (e.g., bacteria and viruses) can help in designing advanced bio-systems with high efficiency and sensitivity to monitor and control the safety and quality of the food products. Although biopolymer-derived emulsion gels had many advantages (cell biocompatibility and biodegradability, source renewability, inherent nutritional composition, *etc*.) over synthetic polymers-based gels, however some properties such as mechanical strength may not fully match. In this regard, the presence of charged species (H^+^ and OH^−^) and different functional groups on biopolymers such as amino, carboxyl, and hydroxyl groups impart huge fabrication potentials that would be utilized in designing gels with superior properties according to the needs.

To further explore the scope of emulsion gels in the food industry, cross-disciplinary studies emphasizing their physicochemical, rheological and tribological, and functional properties are needed to better design effective food formulations. Moreover, detailed investigations both *in vitro* and *in vivo* studies focusing on gel-body interactions specifically gastrointestinal physiology (digestion, biochemical transformations, absorption, and excretion) could help in promoting emulsion gels applications in edible food formulations. For instance, gels with desired sensorial textures and flavors may help in eradicating obesity-related problems by minimizing the intake of fats and sugars and consequently an effective control of cholesterol and glucose metabolism. Moreover, emulsion gels can be used to tailor the sensory perception of bioactive compounds such as phenolic substances and capsaicinoids that had pungent and astringent tastes during oral processing.

## Author Contributions

A and JX: conceptualization. A, LL, and HJ: writing-original draft preparation. A and JX: writing-review and editing. JX: supervision and resources, and funding acquisition. All the authors have read, revised, and agreed to manuscript publication.

## Funding

The authors would like to recognize the financial support of the Guangdong Introducing Innovative and Entrepreneurial Teams under Grant (Project No. 2019ZT08N291) and the Foreign Technology Cooperation Plan of Guangzhou (No. 201907010031).

## Conflict of Interest

The authors declare that the research was conducted in the absence of any commercial or financial relationships that could be construed as a potential conflict of interest.

## Publisher's Note

All claims expressed in this article are solely those of the authors and do not necessarily represent those of their affiliated organizations, or those of the publisher, the editors and the reviewers. Any product that may be evaluated in this article, or claim that may be made by its manufacturer, is not guaranteed or endorsed by the publisher.
